# Supplementary material for: MARVEL: an integrated alternative splicing analysis platform for single-cell RNA sequencing data
Source: Nucleic Acids Res. 2023 Jan 12;51(5):e29. doi: 10.1093/nar/gkac1260 (PMC10018366; doi:10.1093/nar/gkac1260)
Supplement: gkac1260_Supplemental_Files [file gkac1260_supplemental_files.zip › Supplementary Figures 1-14.pdf]

Supplementary Figure 1

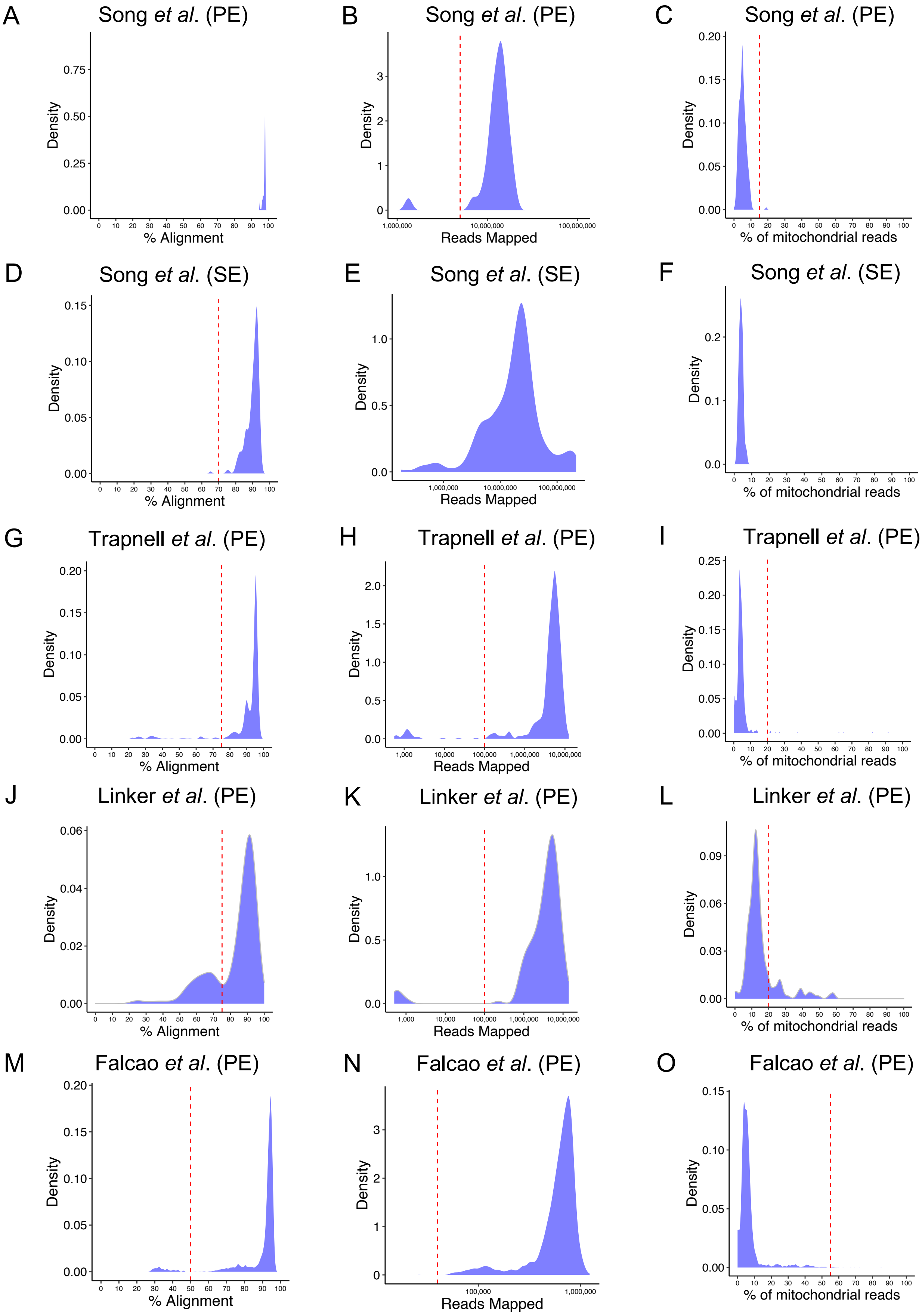

Supplementary Figure 1. QC to identify high-quality cells for downstream analyses for plate-based scRNA-seq datasets. (A-L) Sequencing QC using total reads mapped, alignment rate, and percentage of mitochondrial reads contribution to filter for high-quality cells in **(A-F)** Song *et al.*, **(G-I)** Trapnell *et al.*, **(J-L)** Linker *et al.*, and **(M-O)** Falcao *et al*. dataset. For total reads mapped and alignment rate, cells above the threshold denoted by the red dashed line were considered high-quality. For the percentage of mitochondrial reads contribution, cells below the threshold denoted by the red dashed line were considered high-quality. Only cells meeting all three criteria were included for downstream analyses.

Supplementary Figure 2

A

Description	Alternative splicing event type							Validated?
	SE	MXE	RI	A5SS	A3SS	ALE	AFE	
Only one of both $SJ_{included}$ found			N/A	N/A	N/A	N/A	N/A	No
Only $SJ_{included}$ found			N/A			polyA polyA		No
Only $SJ_{excluded}$ found			N/A					No
Intron overlapping with exonic coordinates	N/A	N/A	Overlapping coordinates	N/A	N/A	N/A	N/A	No
Both $SJ_{included}$ and $SJ_{excluded}$ found			NA			polyA polyA		Yes
$Reads_{included}$ or/and $SJ_{excluded}$ found	N/A	N/A	case 1 case 2 case 3	N/A	N/A	N/A	N/A	Yes

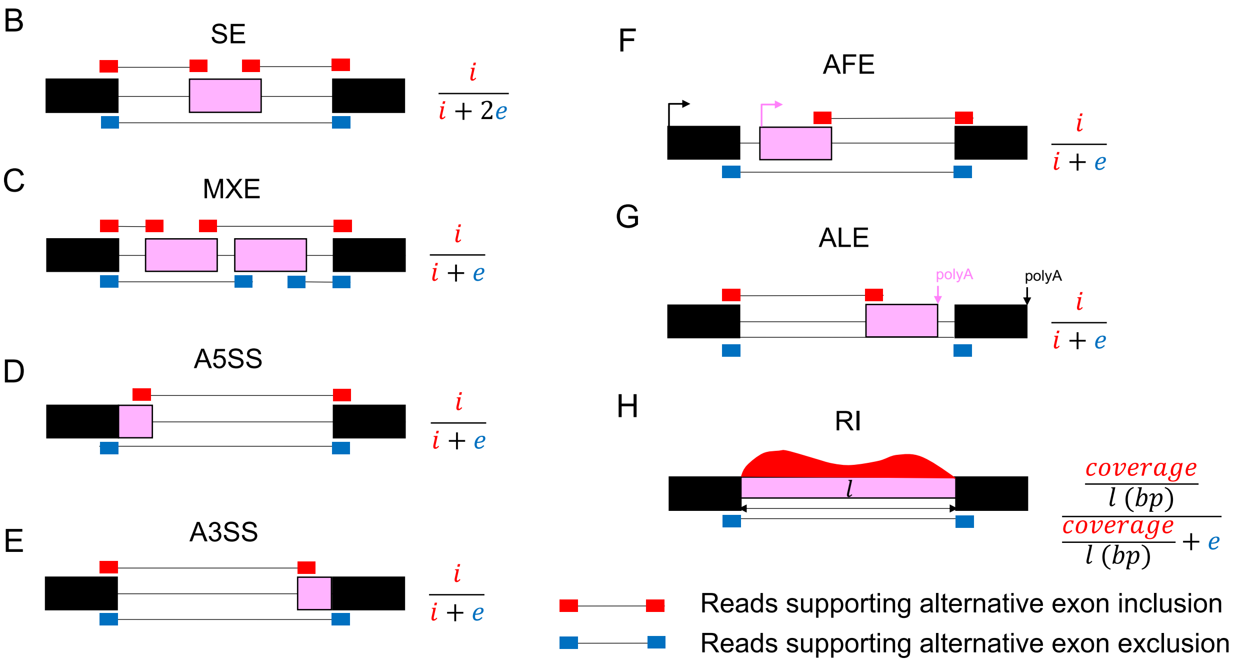

Supplementary Figure 2. Alternative splicing event validation and PSI formulas. (A) Validation of alternative splicing events using splice junction reads to select high-quality alternative splicing events for PSI estimation. **(B-F)** The formulas to estimate the PSI values for **(B)** SE, **(C)** MXE, **(D)** A5SS, **(E)** A3SS, **(F)** AFE, **(G)** ALE, and **(H)** RI. i denotes the number of splice junction reads supporting the alternative exon(s) in pink. e denotes the number of splice junction reads supporting the constitutive exon(s) in black but skipping the alternative exon(s). $coverage$ denotes the intron coverage. l denotes the intron length in base-pair (bp).

Supplementary Figure 3

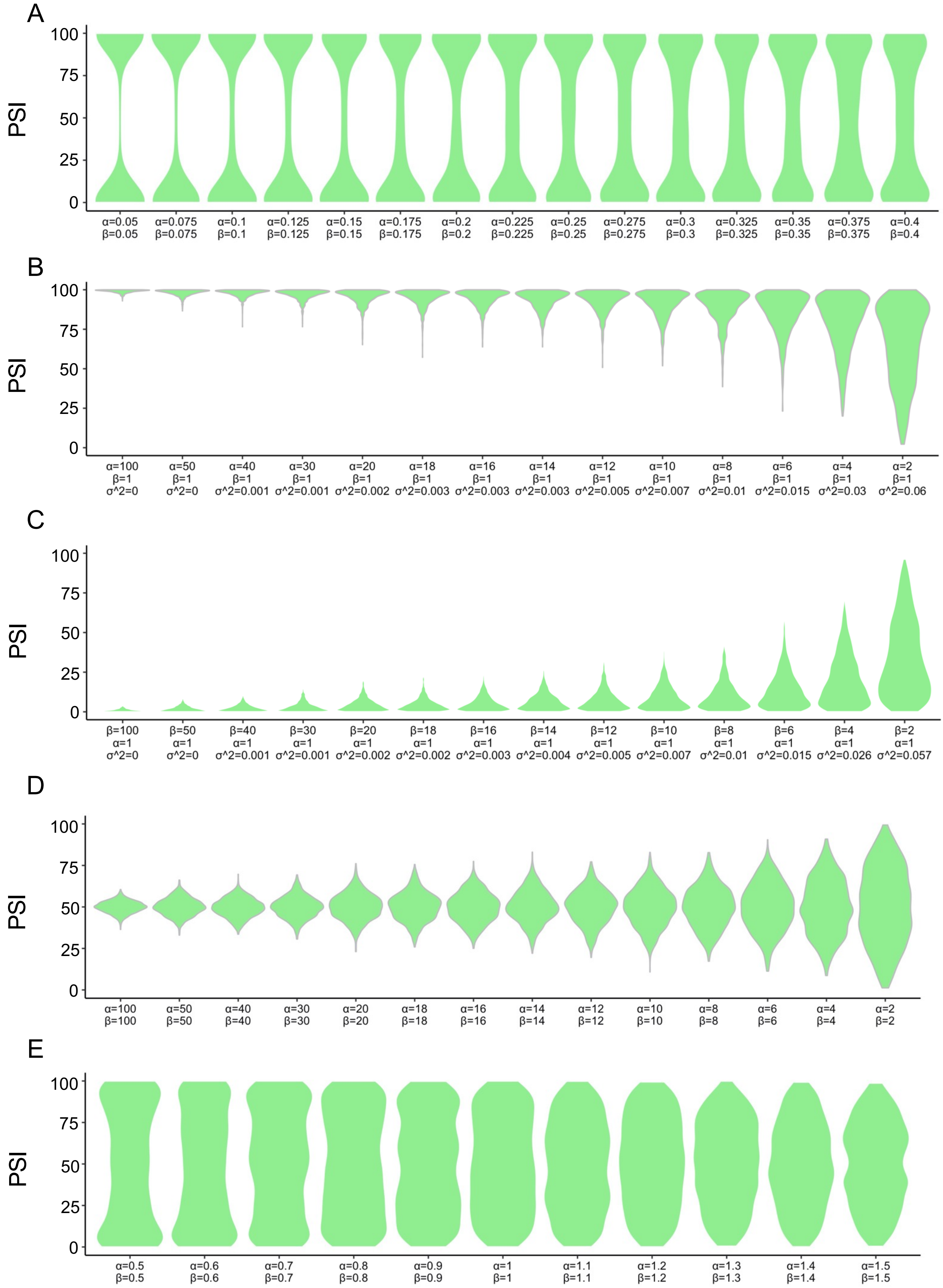

Supplementary Figure 3. The simulation of PSI values in different PSI distributions for each modality class. (A-E) PSI distributions corresponding to **(A)** bimodal, **(B)** included, **(C)** excluded, **(D)** middle, and **(E)** multimodal. The PSI distributions were modeled using the beta distribution, and the corresponding α and β parameters were estimated using the maximum likelihood approach.

Supplementary Figure 4

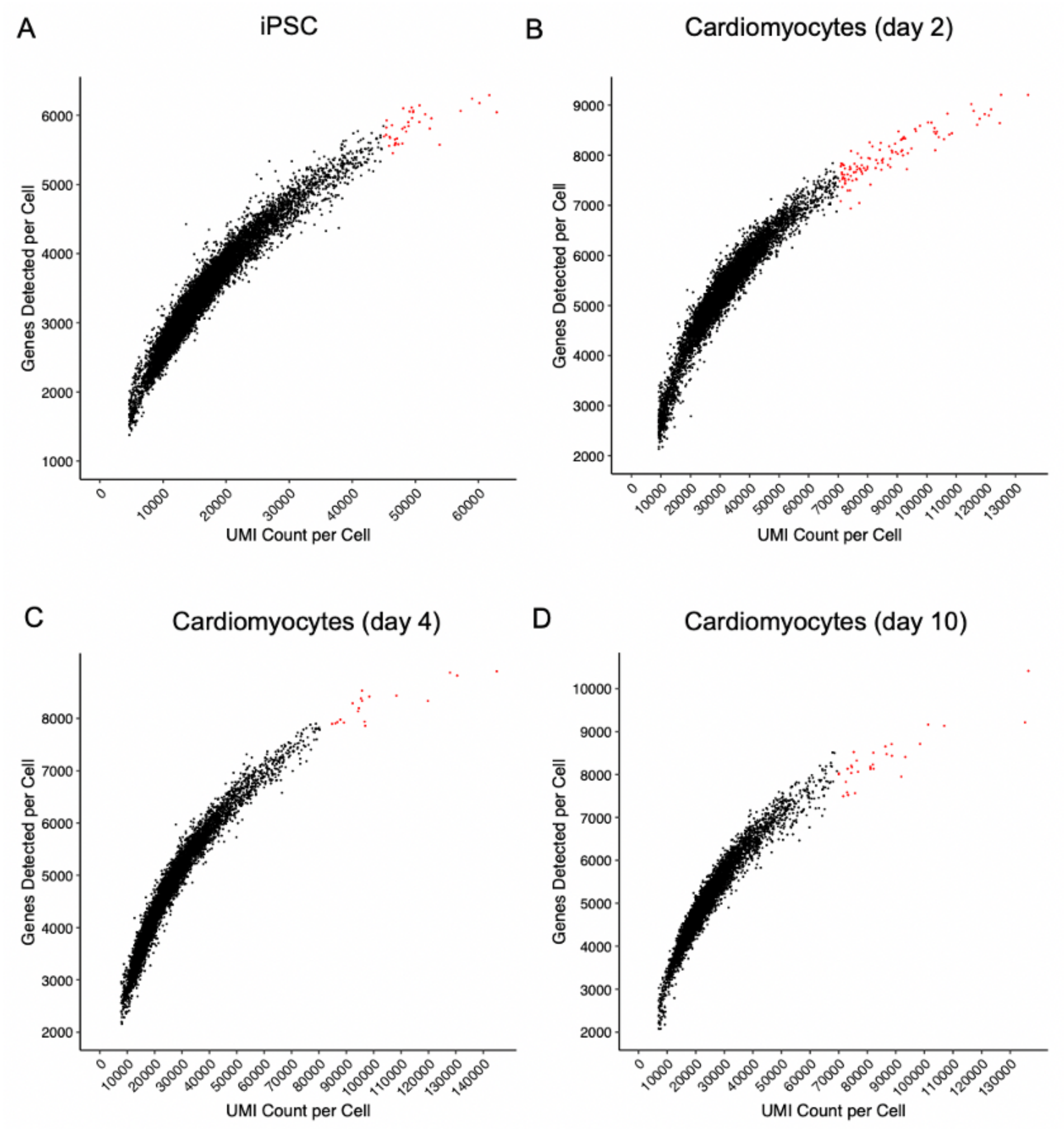

Supplementary Figure 4. QC to identify high-quality cells for downstream analyses for the droplet-based scRNA-seq dataset. (A-D) QC of single cells based on the number of genes detected and number of UMIs for **(A)** iPSCs, and cardiomyocytes at days **(B)** 2, **(C)** 4, and **(D)** 10. The red cells were excluded from downstream analyses based on UMI counts and the number of detected genes. UMI: unique molecular identifier.

Supplementary Figure 5

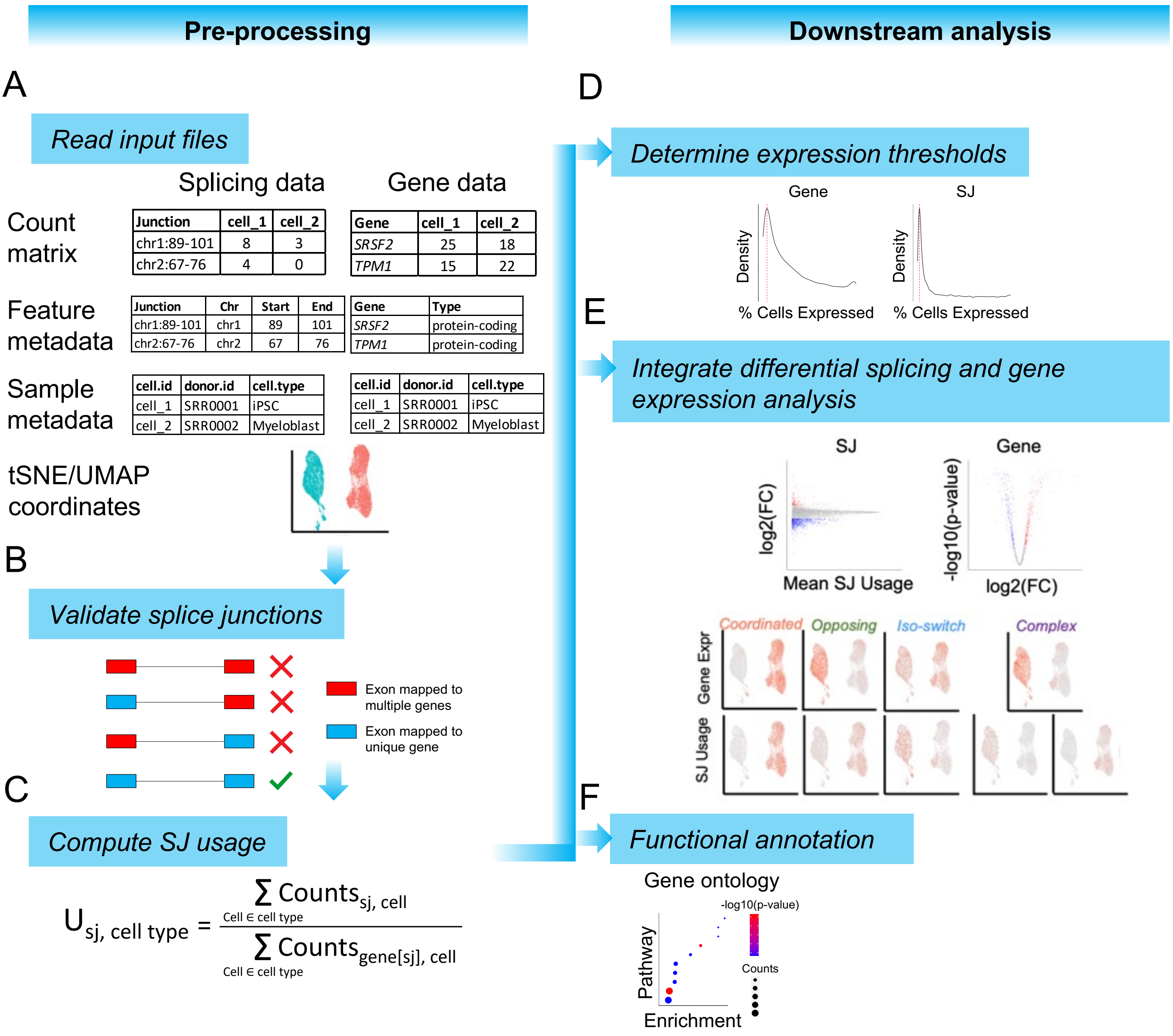

Supplementary Figure 5. MARVEL workflow for single-cell alternative splicing analysis in a droplet-based scRNA-seq dataset. (A-C) Pre-processing of splice junction and gene expression data. **(A)** Inputs for MARVEL include splice junction and gene count matrix, normalized gene expression matrix, and splice junction, gene and sample metadata, and dimension reduction coordinates. **(B)** Only splice junctions, in which both exons are mapped to the same unique gene are retained. **(C)** For a given cell population, the splice junction usage is computed for the high-quality splice junctions identified in **(B)** as the total splice junction counts divided by the total corresponding gene counts. **(D-E)** Downstream analyses using the computed splice junction usage and gene expression values. **(D)** Splice junction and gene expression distributions across a given cell population. Red dotted lines correspond to the percentage of cells in which most splice junctions and genes are expressed and can be used as thresholds for sub-setting splice junctions and genes for differential analysis. **(E)** Integrative differential splice junction and gene expression analysis allows for investigating changes in splice junction usage relative to changes in gene expression across different cell populations. **(F)** Pathway enrichment analysis to identify gene sets that are coordinatedly spliced. iPSC: Induced pluripotent stem cells.

Supplementary Figure 6

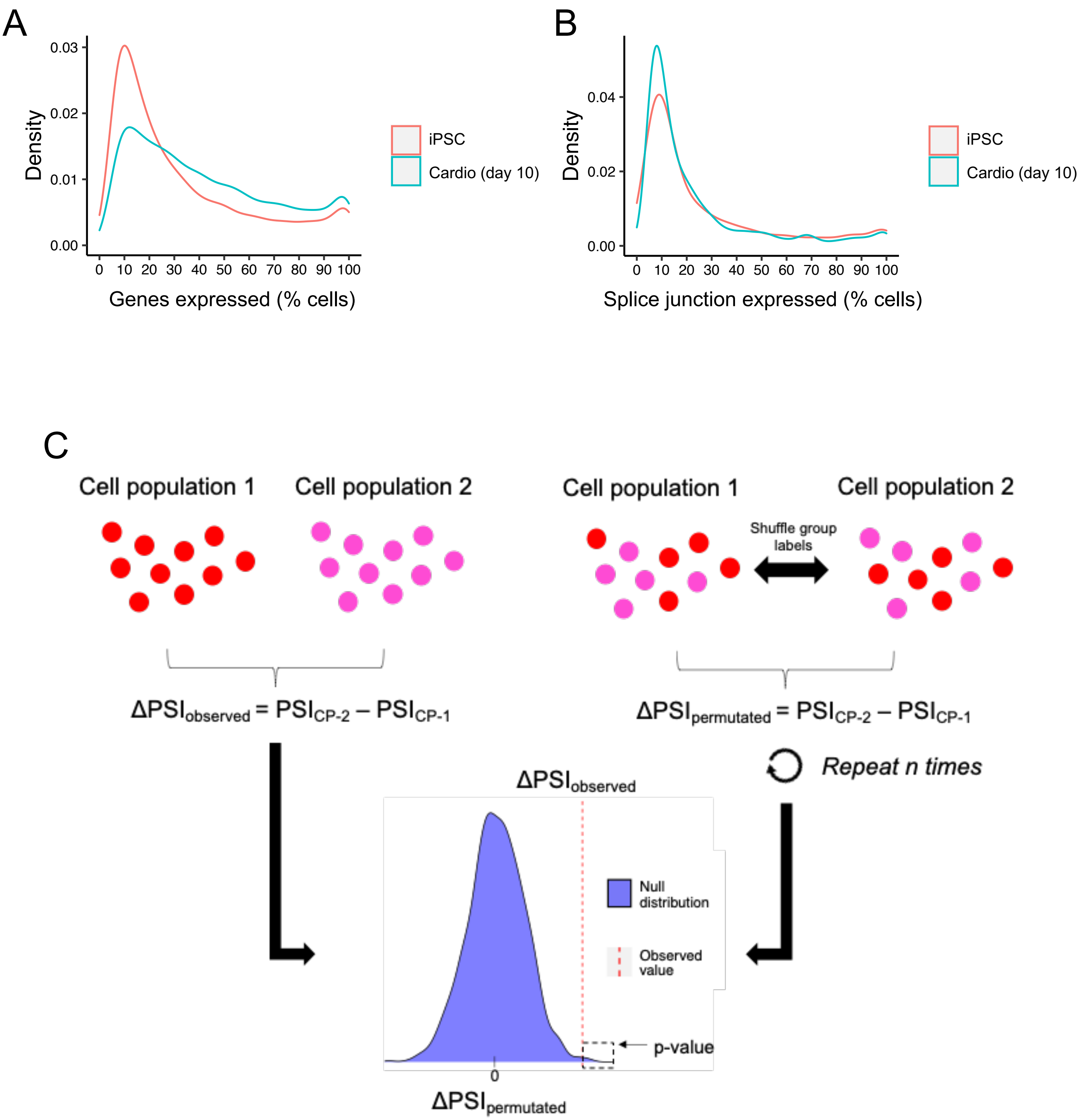

Supplementary Figure 6. Gene selection and differential splicing analysis for a droplet-based dataset. (A) The percentage density plot of cells in which a gene is expressed in each cell type. **(B)** The percentage density plot of cells in which a splice junction is expressed in each cell type. **(C)** A proposed procedure for the permutation approach for assessing differentially spliced junctions between two cell populations. PSI values for cell populations 1 and 2 are computed. Then, the observed differences in the PSI values between two cell populations ($\Delta PSI_{observed}$) are noted. The cell-type labels of single cells are shuffled, and differences in the PSI values between populations are noted and iterated 100 times ($\Delta PSI_{permutated}$). These permutated differences in the PSI values will form the null distribution. Then, the observed differences in the PSI values between the cell populations ($\Delta PSI_{observed}$) are compared against the null distribution to obtain P-values.

Supplementary Figure 7

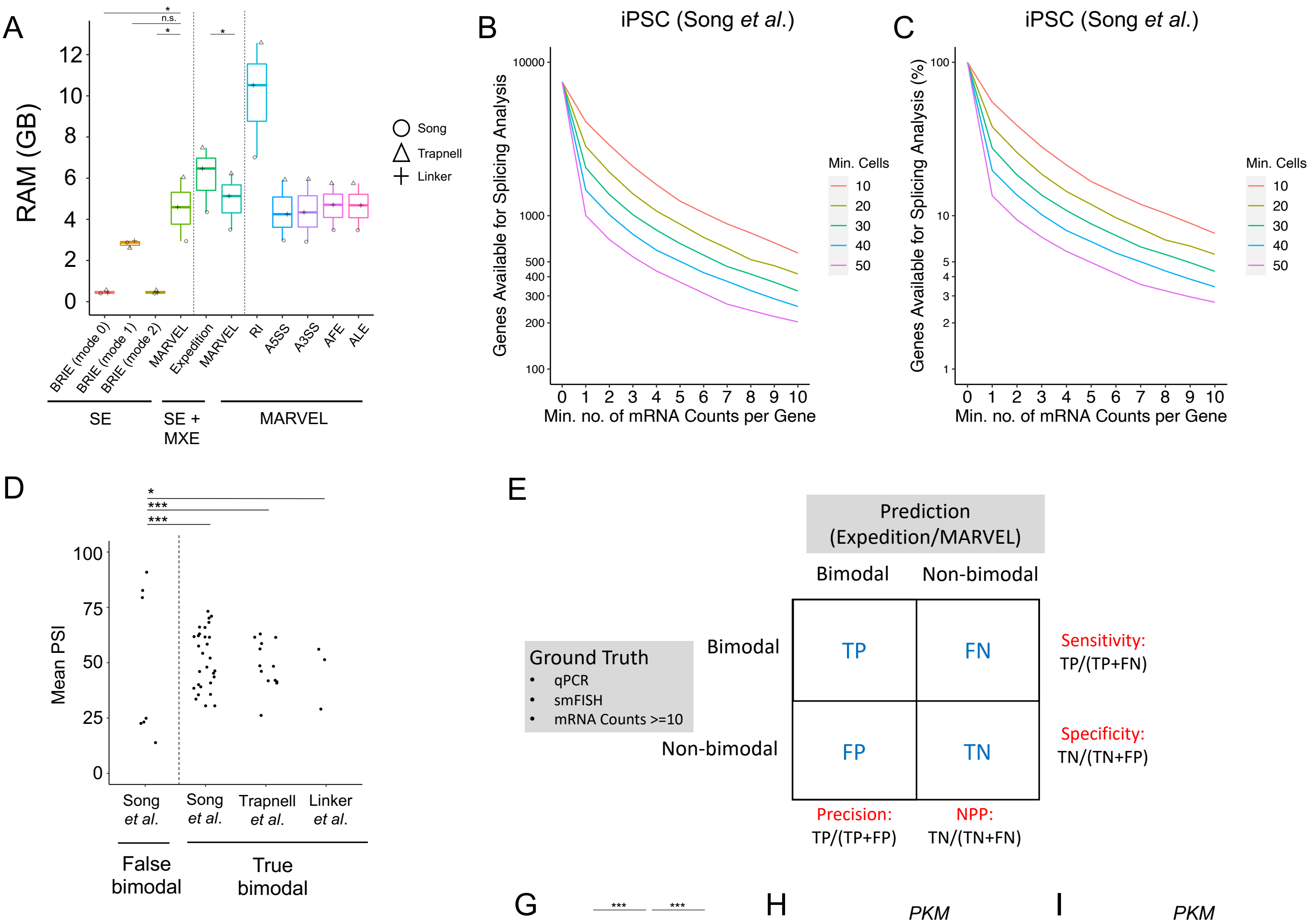

F

Expedition

		Bimodal	Non-bimodal
Ground truth	Bimodal	43	2
	Non-bimodal	203	17,056

MARVEL

		Bimodal	Non-bimodal
Ground truth	Bimodal	44	1
	Non-bimodal	9	17,250

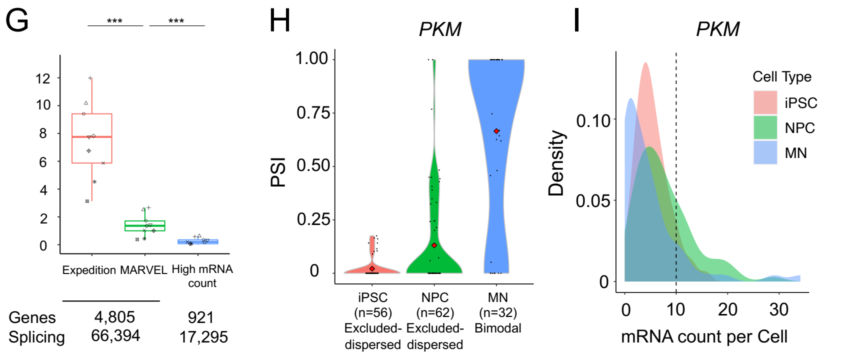

Supplementary Figure 7. Benchmarking MARVEL against existing computational tools for estimating PSI values and modality assignment. (A) The use of RAM in GB to compute PSI values for 1,000 splicing events for each splicing event type in three datasets. **(B-C)** In iPSCs, the **(B)** number or **(C)** percentage of genes for alternative splicing analysis at different mRNA count thresholds and the different minimum number of cells thresholds (sample size). **(D)** The average PSI values of false and true bimodal distributions. **(E-F)** The confusion matrix comparing the number of bimodal and non-bimodal classifications assigned by Expedition and MARVEL compared to that of the ground truth consisting of 17,304 false and true bimodal distributions. **(G)** The percentage of alternative splicing events assigned as bimodal distribution by Expedition, MARVEL, and high mRNA count approach. **(H)** The PSI distribution for *PKM* alternative splicing event in iPSCs, NPCs, and MNs. **(I)** The mRNA count distribution for *PKM* in iPSCs, NPCs, and MNs. RAM: Random Access Memory; GB: Gigabyte. *** FDR < 0.01 ** FDR < 0.05 * FDR < 0.1.

Supplementary Figure 8

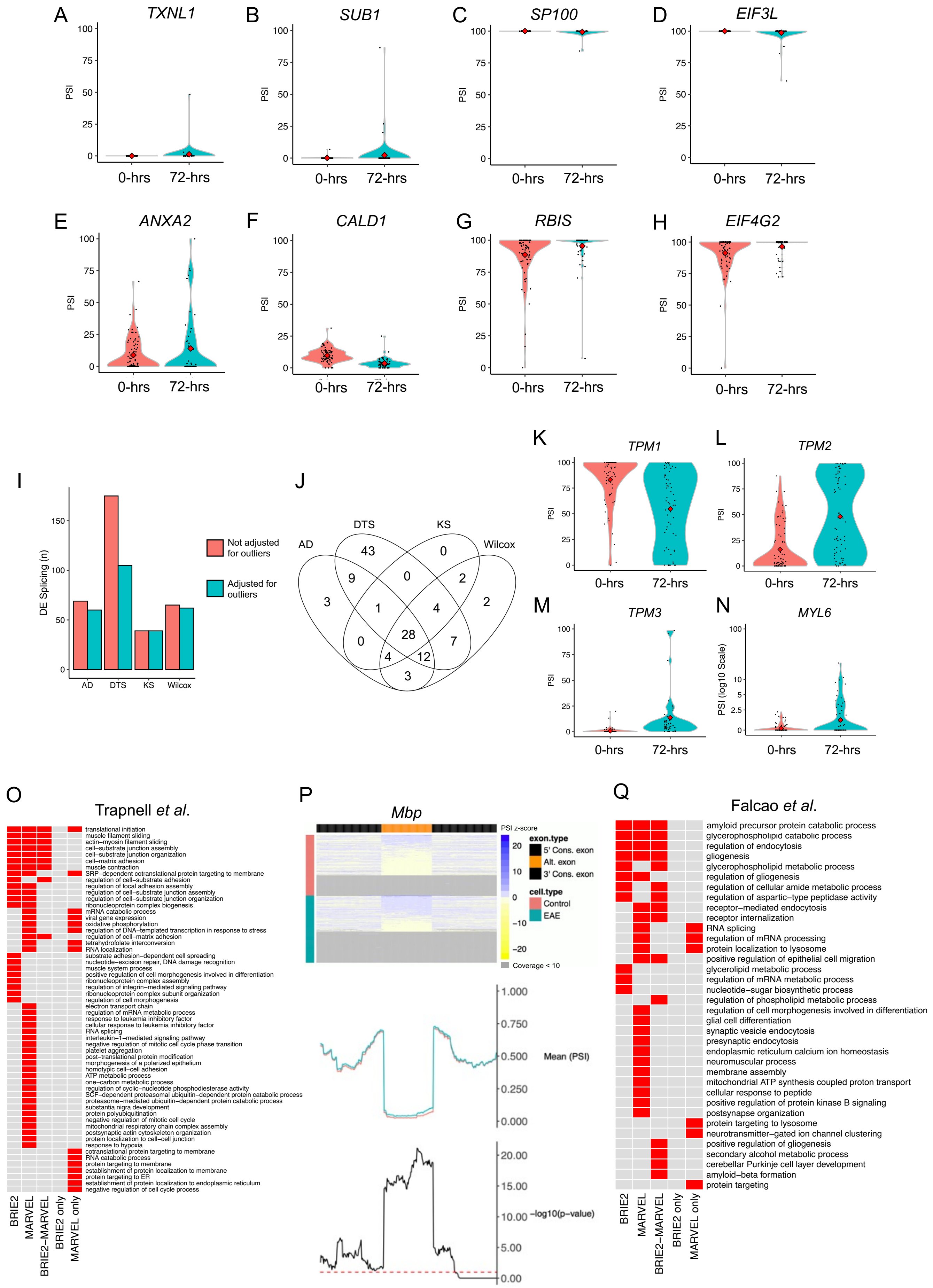

Supplementary Figure 8. Benchmarking MARVEL against existing computational tools for differential splicing analysis. **(A-D)** Representative examples of splicing events, identified as differentially spliced by DTS, driven by the small number of cells (outliers) with PSI values of **(A-B)** >0 or **(C-D)** <1 when the modality change is from excluded to excluded or included to included, respectively. **(E-H)** Representative examples of splicing events that were considered as differentially spliced when the number of cells with PSI values of **(E-F)** >0 or **(G-H)** <1 when in either cell group is above a user-defined threshold, in this study is 10, when the modality change is from included to included or excluded to excluded, respectively. **(I)** The number of differentially spliced events detected between 72- vs. 0-hrs myoblast before and after removing events driven by outlier cells. **(J)** The number of differentially spliced events detected by AD, DTS, KS, and Wilcoxon rank-sum tests between 72- vs. 0-hrs myoblast. **(K-N)** Representative examples of muscle-related differentially spliced genes detected by MARVEL. **(O)** Identified pathways enriched among differentially spliced genes between 72- vs. 0-hrs myoblast among differentially spliced events identified by "BRIE2" (all spliced genes identified by BRIE2), "MARVEL" (all spliced genes identified by MARVEL), "BRIE2-MARVEL overlap" (spliced genes detected by both BRIE2 and MARVEL), "BRIE2-only" (spliced genes identified by BRIE2 but not MARVEL), and "MARVEL-only" (spliced genes identified by MARVEL but not BRIE2). **(P)** The visual validation of *Mbp* exon 2 shows differentially spliced between EAE and control mice by MARVEL. This exon2 was also previously validated using qPCR by the original study. **(Q)** Identified pathways enriched among differentially spliced genes between EAE and control mice among differentially spliced events identified by "BRIE2" (all spliced genes identified by BRIE2), "MARVEL" (all spliced genes identified by MARVEL), "BRIE2-MARVEL overlap" (spliced genes detected by both BRIE2 and MARVEL), "BRIE2-only" (spliced genes identified by BRIE2 but not MARVEL), and "MARVEL-only" (spliced genes identified by MARVEL but not BRIE2). AD: Anderson-Darling; DTS: D Test Statistic; EAE: experimental autoimmune encephalomyelitis; KS: Kolmogorov-Smirnov; hrs: hours; PSI: percent spliced-in; qPCR: quantitative polymerase chain reaction.

Supplementary Figure 9

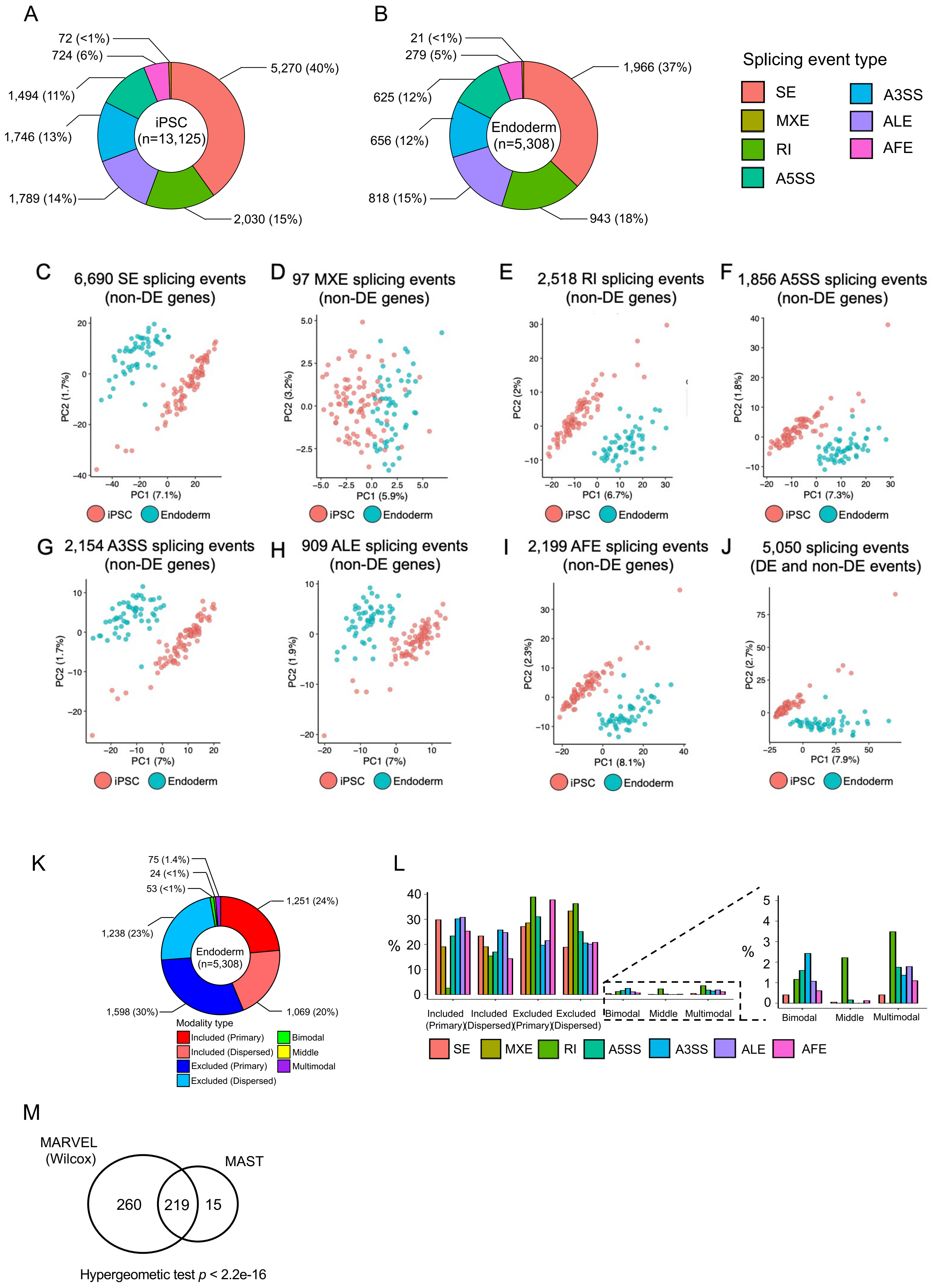

Supplementary Figure 9. Application of MARVEL on iPSCs differentiated to endoderm cells. (A-B) The proportion of expressed alternative splicing event types in **(A)** iPSCs and **(B)** endoderm cells. **(C-J)** Dimension reduction analysis with PCA using **(C)** SE, **(D)** MXE, **(E)** RI, **(F)** A5SS, **(G)** A3SS, **(H)** ALE, and **(I)** AFE splicing events of non-differentially expressed genes, and **(J)** all splicing events regardless of if there were differentially spliced or not. **(K)** The proportion of each modality class in endoderm cells. **(L)** The proportion of each modality class by splicing event type in endoderm cells. **(M)** Number of overlapping differentially expressed genes among differentially spliced genes identified using Wilcoxon rank-sum test and MAST. A3SS: alternative 3' splice site; A5SS: alternative 5' splice site; AFE: alternative first exon; ALE: alternative last exon; MXE: mutually exclusive exons; RI: retained-intron; SE: skipped-exon.

Supplementary Figure 10

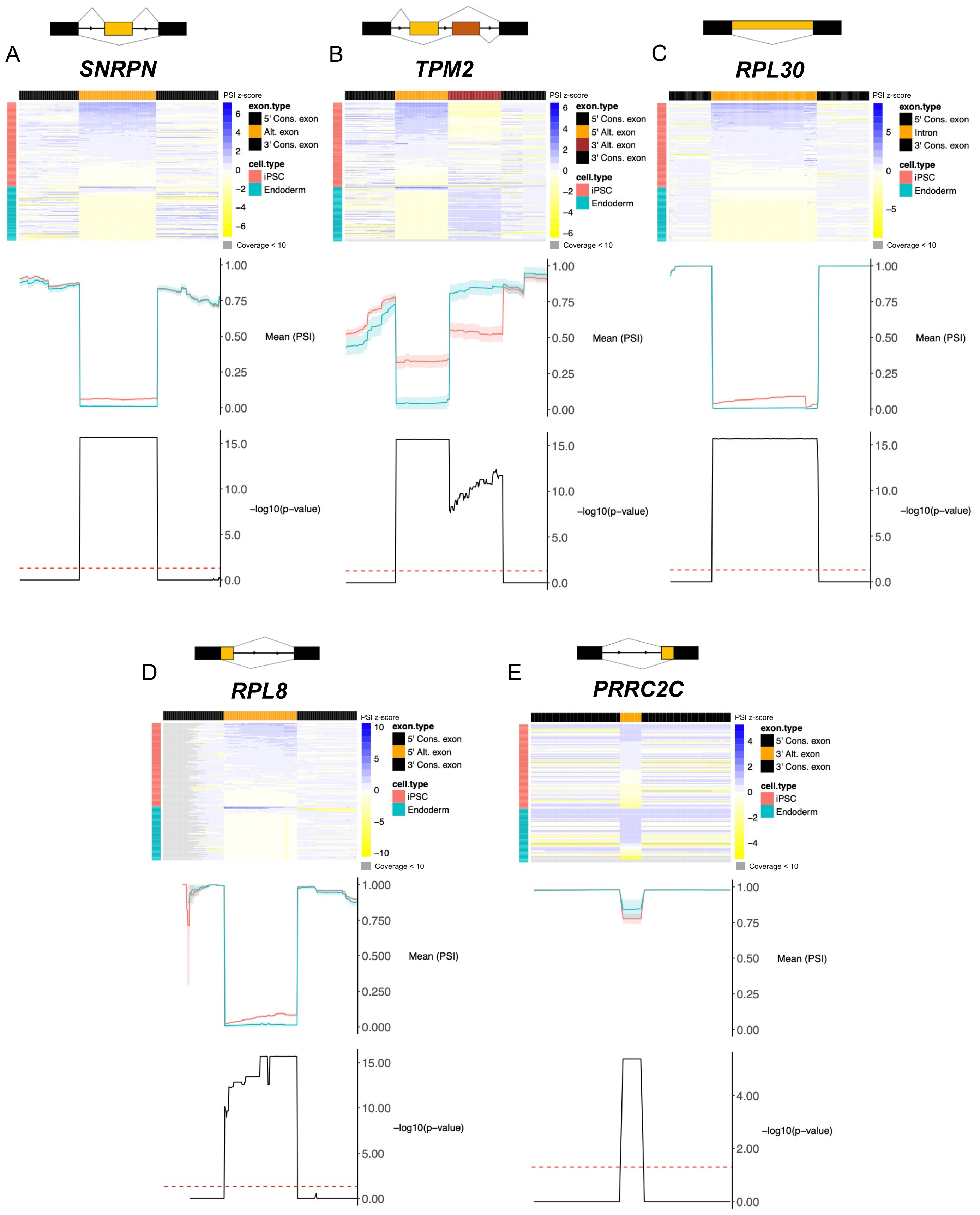

Supplementary Figure 10. Visual validation of differential alternative splicing events detected by MARVEL using VALERIE. (A-E) Representative examples of genes for each splicing event, **(A)** SE, **(B)** MXE, **(C)** RI, **(D)** A5SS, and **(E)** A3SS visually inspected using VALERIE. A3SS: alternative 3' splice site; A5SS: alternative 5' splice site; MXE: mutually exclusive exons; RI: retained-intron; SE: skipped-exon.

Supplementary Figure 11

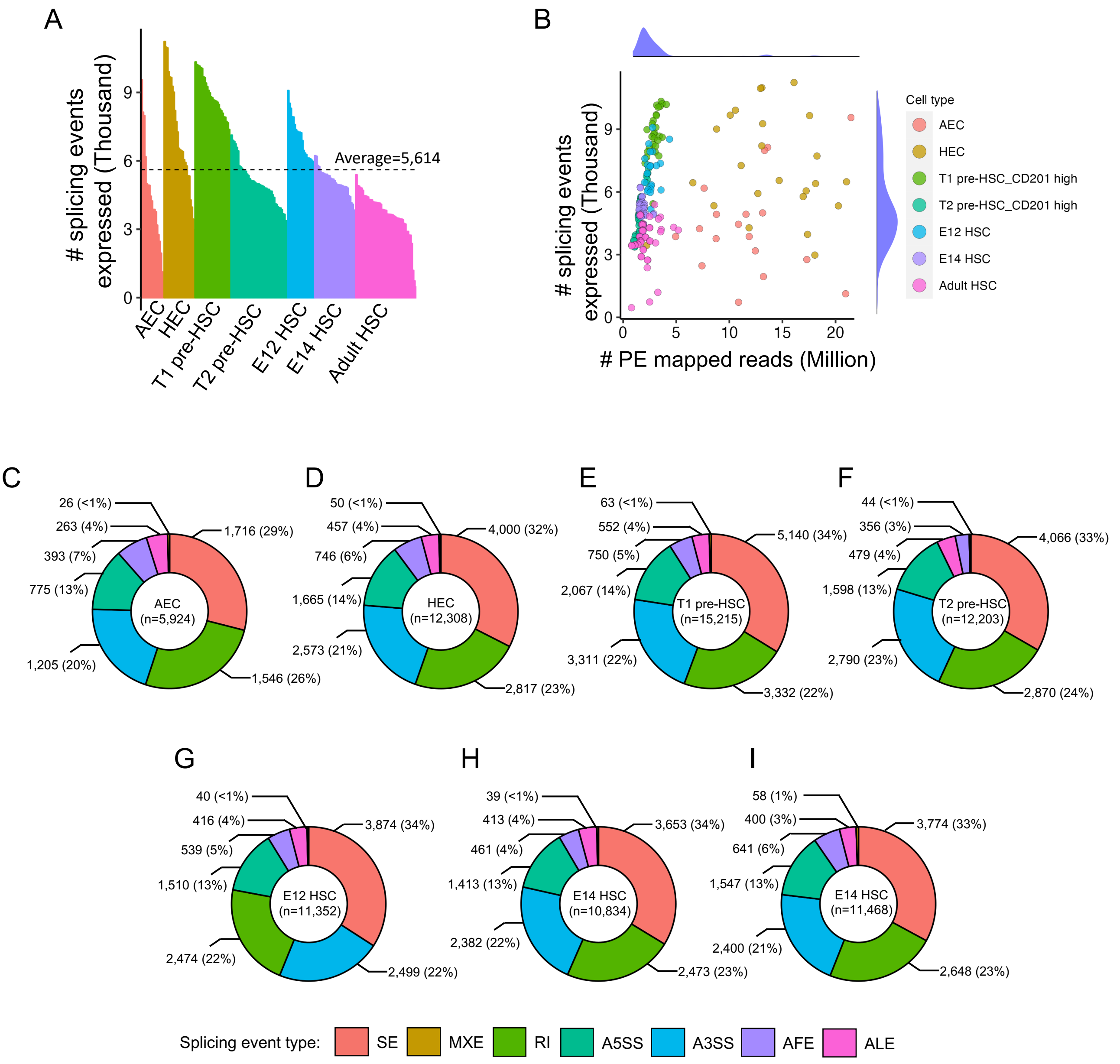

Supplementary Figure 11. Splicing events expressed in single cells derived during mouse endothelial-to-hematopoietic stem cell (HSC) transition. **(A)** Number of splicing events expressed in each cell. **(B)** Number of splicing events expressed vs. sequencing depth for each cell. **(C-I)** Number of expressed events stratified by splicing event type for each cell population. AEC: aortic endothelial cells; HEC: Hemogenic endothelial cells.

Supplementary Figure 12

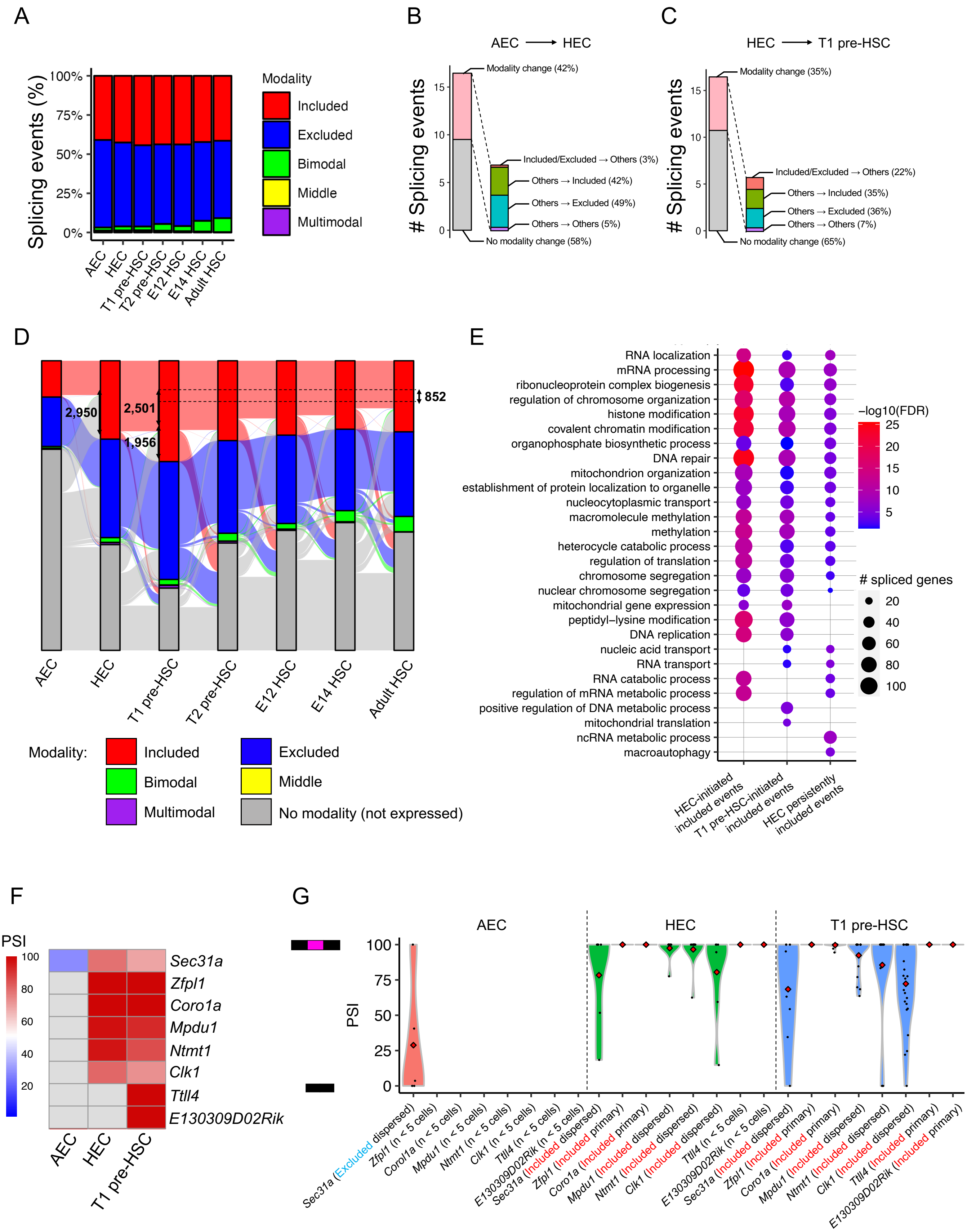

Supplementary Figure 12. Modality analysis during mouse endothelial-to-hematopoietic stem cell (HSC) transition. (A) Proportion of modality categories in each cell population. **(B-D)** Rate of modality change during **(B)** AEC to HEC transition, **(C)** HEC to T1 pre-HSC transition, and **(D)** transition across all cell populations from AEC to adult HSC. **(E)** Top 15 pathways enriched among genes that constitute the HEC- and T1 pre-HSC-initiated HSCs, and HEC-presistently included splicing events. (F-G) Splicing profile of HEC- and T1 pre-HSC-initiated HSCs, and HEC-presistently included splicing events that were experimentally validated using FISH in the original publication (Wang *et al*., 2022)**.**

Supplementary Figure 13

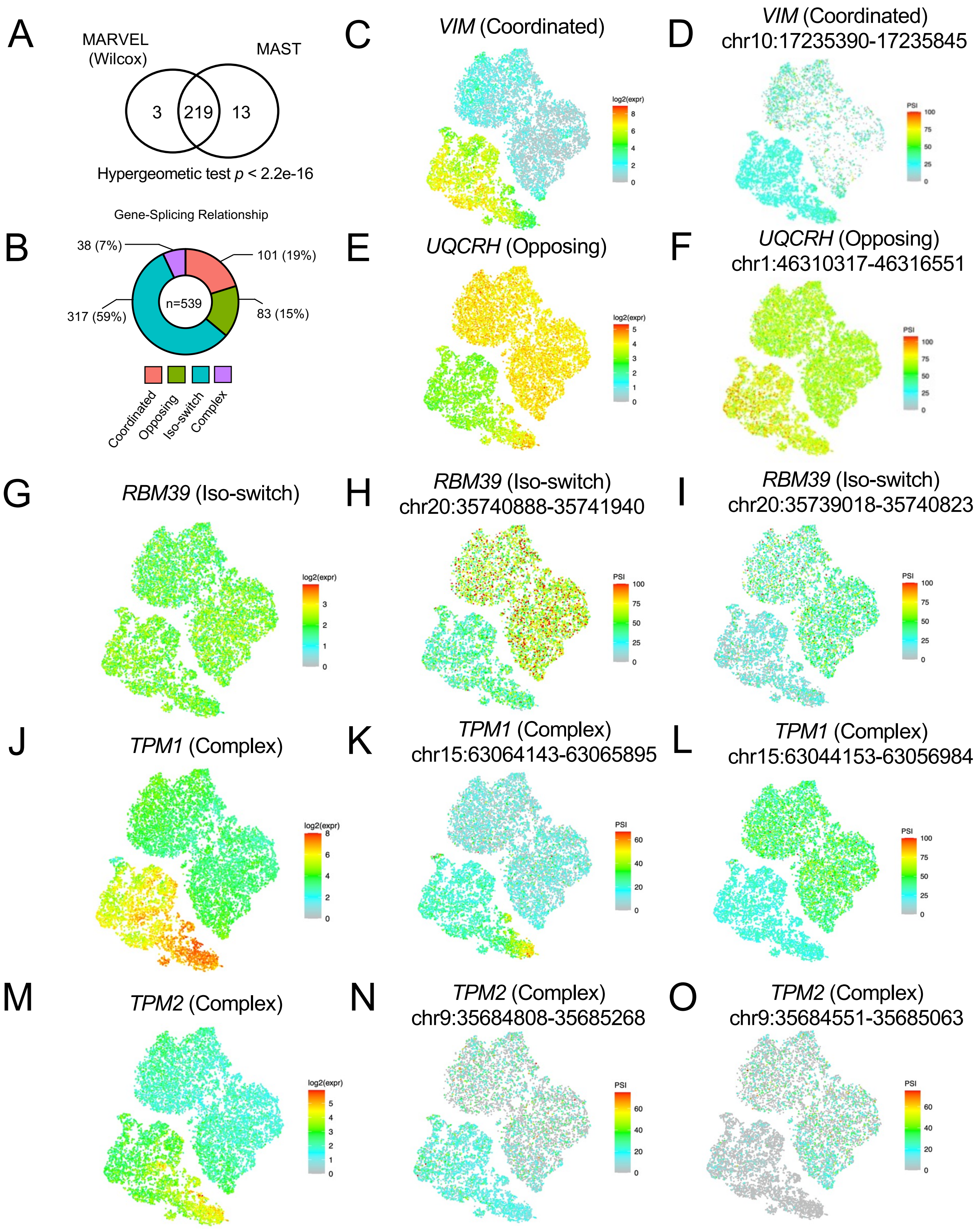

Supplementary Figure 13. Gene expression and splice junction usage relationship of differentially spliced genes identified from iPSCs differentiated to day-10 cardiomyocytes. (A) Number of overlapping differentially expressed genes among differentially spliced genes identified using Wilcoxon rank-sum test and MAST. **(B)** The proportion of each gene-splicing relationship class, i.e., change in average gene expression value relative to change in average splice junction usage value for the corresponding splice junction from iPSCs differentiated to cardiomyocytes. **(C-O)** Representative gene expression and corresponding splice junctions for each gene-splicing relationship class, **(C-D)** coordinated, **(E-F)** opposing, **(G-I)** isoform switching, and **(J-O)** complex. Gene expression levels are indicated by the log2 scale. The splicing rate is indicated by the PSI scale. The genomic coordinates are also indicated for the respective splice junctions.

Supplementary Figure 14

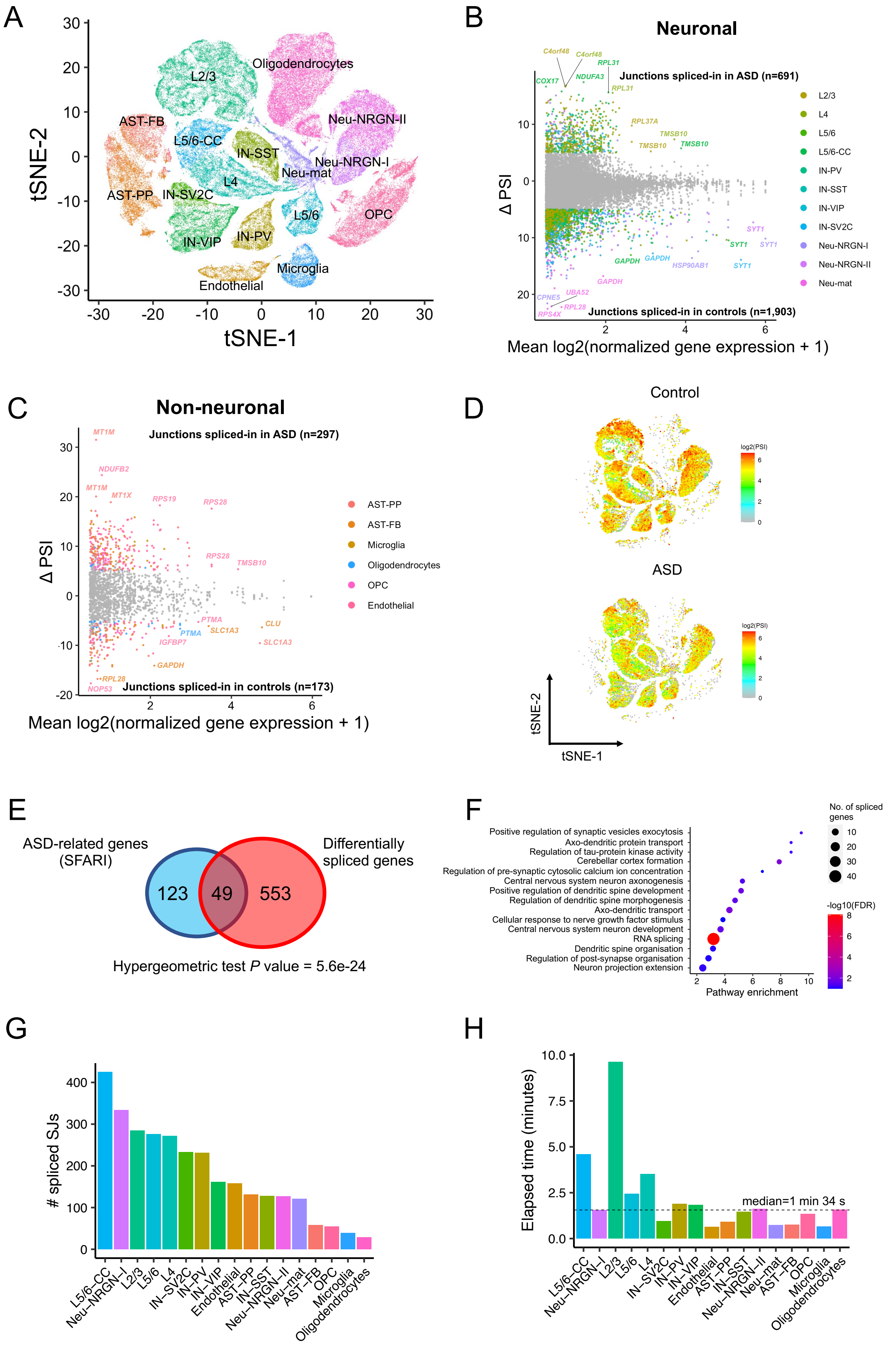

Supplementary Figure 14. Differential splicing analysis of single cells derived from brain tissues of 15 autism spectrum disorder (ASD) patients and 16 healthy controls from Velmeshev *et al*. (A) The cell populations identified by the original study and included for analysis here. **(B-C)** Differential splicing analysis and top differentially spliced junctions annotated for **(B)** neuronal and **(C)** non-neuronal cell types. **(D)** Splice junction chr12:78865110-78977798 belonging to *SYT1*, a canonical marker gene for excitatory neurons, was significantly spliced-out in ASD patients relative to controls. **(E)** Overlap of differentially spliced genes from this analysis with previously reported ASD-related genes. **(F)** Pathway enrichment of differentially spliced genes. **(G)** Overall number of significantly spliced junctions by cell type. **(H)** Processing time taken for differential splicing analysis for each cell type computed using MacBook Pro with in-built 2 GHz Quad-Core Intel Core i5 processor and 16 GB 3733 MHz LPDDR4X memory.
