# Supplementary material for: MARVEL: an integrated alternative splicing analysis platform for single-cell RNA sequencing data
Source: Nucleic Acids Res. 2023 Jan 12;51(5):e29. doi: 10.1093/nar/gkac1260 (PMC10018366; doi:10.1093/nar/gkac1260)
Supplement: gkac1260_Supplemental_Files [file gkac1260_supplemental_files.zip › Supplementary Table 1.docx]

**Supplementary Table 1. Analysis features provided by MARVEL compared to published single-cell alternative splicing platforms.**

| **Feature** | **BRIE** | **Expedition** | **MARVEL** | **SCATS** | **DESJ-detection** | **VALERIE** |
| --- | --- | --- | --- | --- | --- | --- |
| PSI estimation (exon level) |  |  |  |  |  |  |
| Skipped exon | / | / | / | / | x | / |
| Mutually exclusive exons | x | / | / | x | x | / |
| Retained intron | x | x | / | x | x | / |
| Alternative 5' splice site | x | x | / | x | x | / |
| Alternative 3' splice site | x | x | / | x | x | / |
| Alternative first exon | x | x | / | x | x | x |
| Alternative last exon | x | x | / | x | x | x |
| PSI estimation (splice junction level) | x | x | / ^a^ | x | / | x |
| Incorporate UMIs in PSI estimation ^b^ | x | x | x | / | x | x |
| Modality classification |  |  |  |  |  |  |
| Main modalities | x | / | / | x | x | x |
| Sub-modalities | x | x | / | x | x | x |
| False bimodal correction | x | x | / | x | x | x |
| Dimension reduction analysis | / | x | / | x | x | x |
| Differential expression analysis |  |  |  |  |  |  |
| Differential splicing analysis | / | x | / | / | / | / |
| Differential gene expression analysis | x | x | / | x | x | x |
| Functional annotation |  |  |  |  |  |  |
| Gene ontology | x | x | / | x | x | x |
| Nonsense-mediated decay prediction | x | x | / | x | x | x |
| RNA velocity | / | x | x | x | x | x |
| Visual validation | x | x | x | x | x | / |
| Processing time (relative to MARVEL) ^c^ | 2.8 ^d^ | 24.9 | 1 (reference) | NR | NR | NR |
| RNA-sequencing platform |  |  |  |  |  |  |
| Plate-based (e.g., Smart-seq2) | / | / | / | / | / | / |
| Droplet-based (e.g., 10x Genomics) | x | x | / | x | x | x |

^a^ Droplet-based; ^b^ Plate-based;

^c^ Median processing time in seconds to compute the PSI values of 1,000 splicing events as per Figure 2E; ^d^ BRIE (mode 2)

NR: Not reported by the original study
